# Knockdown of S100A11 expression suppresses ovarian cancer cell growth and invasion

**DOI:** 10.3892/etm.2015.2257

**Published:** 2015-02-03

**Authors:** YOUQING LIU, XIAOBING HAN, BAOAN GAO

**Affiliations:** 1 Department of Obstetrics and Gynecology, Anhui Provincial Hospital Affiliated to Anhui Medical University, Hefei, Anhui 230001, P.R. China; 2 Department of Obstetrics and Gynecology, The First Affiliated Hospital, Medical School of Xi’an Jiaotong University, Xi’an, Shaanxi 710061, P.R. China; 3 Department of Respiratory Medicine, The First College of Clinical Medical Science, China Three Gorges University and Yichang Central People’s Hospital, Yichang, Hubei 443003, P.R. China

**Keywords:** S100A11, growth, invasion, migration, ovarian cancer

## Abstract

As a member of the S100 protein family, S100A11 expression is often upregulated in human cancer tissues. Numerous studies have demonstrated that S100A11 plays an important role in the progression of cancer. However, the function of S100A11 in ovarian cancer remains elusive. In the present study, the expression levels of S100A11 were found to be significantly increased in ovarian cancer cells. Subsequently, the expression of S100A11 in ovarian cancer HO8910 cells was knocked down using short hairpin (sh)RNA in order to investigate the biological effects of S100A11 on the progression of the disease. The results demonstrated that knockdown of S100A11 by shRNA inhibited the proliferation, anchorage-independent growth, invasion and migration of HO8910 cells. In addition, knockdown of S100A11 increased the expression of E-cadherin and decreased the expression of Snail in HO8910 cells. Collectively, these results indicated that S100A11 was able to promote the growth, invasion and migration of ovarian cancer cells. Therefore, S100A11 may serve as a potential molecular target for the diagnosis and treatment of ovarian cancer.

## Introduction

Ovarian cancer is a common gynecological malignancy and remains one of the leading causes of cancer-related mortality among females (1). Proliferation, invasion and metastasis are crucial processes in the development of ovarian cancer, and metastasis occurs prior to diagnosis in the majority of patients with ovarian cancer. The expression of a variety of proteins can change during the progression of cancer. Thus, investigating the functions of these proteins may offer novel targets for the diagnosis and treatment of ovarian cancer (2).

S100 proteins are a group of low molecular weight (10–12 kDa) acidic proteins that belong to the largest family of EF-hand calcium-binding proteins and are only expressed in vertebrates (3). S100A11, also known as S100C or calgizzarin, is an important member of the S100 family (4). Overexpression of S100A11 has been reported in a number of cancers, including papillary thyroid carcinoma (5), colon (6), pancreatic (7) and breast cancer (8). A previous study demonstrated that increased S100A11 expression is correlated with the metastasis of gastric cancer and poor overall disease prognosis (9). However, decreased expression of S100A11 has been reported in bladder cancer, and downregulation of S100A11 has been associated with bladder cancer progression (10). The role of S100A11 in ovarian cancer remains unclear. Therefore, the aim of the present study was to analyze the levels of S100A11 in ovarian cancer cells. In addition, the effects of S100A11 knockdown on ovarian cancer cell growth and invasion were investigated in order to determine the function of S100A11 in ovarian cancer progression.

## Materials and methods

### Antibodies

An antibody targeting E-cadherin (rabbit polyclonal; 1:1,000; cat. no. sc-21791) was purchased from Santa Cruz Biotechnology, Inc. (Dallas, TX, USA). An antibody targeting Snail (mouse monoclonal; 1:1,000; cat. no. 3895) was obtained from Cell Signaling Technology, Inc. (Danvers, MA, USA), while antibodies targeted against S100A11 (mouse monoclonal; 1:1,000; cat. no. WH0006282M1) β-actin (mouse monoclonal; 1:1,000; cat. no. A3853) were obtained from Sigma-Aldrich (St. Louis, MO, USA).

### Cell culture

Cell lines were purchased from the Cell Bank of the Chinese Academy of Sciences (Shanghai, China). The human ovarian surface epithelial cell line, IOSE144, was maintained in MCDB105 medium containing L-glutamine, HEPES (25 mM) and 10% fetal bovine serum (FBS) (all Sigma-Aldrich). Human ovarian cancer cell lines, A2780 and SKOV3, were maintained in Dulbecco’s modified Eagle’s medium containing 10% FBS, while the human ovarian cancer cell lines, OVCAR3 and HO8910, were maintained in RPMI 1640 medium containing 10% FBS. Cells were stored at 37°C in a humidified atmosphere of 5% CO_2_.

### RNA interference of S100A11

An S100A11 short hairpin (sh)RNA vector was designed and synthesized by Shanghai GenePharma Co., Ltd (Shanghai, China), with a target sequence of 5′-GGATGGTTATAACTACACT-3′. A scramble shRNA vector was used as a negative control. HO8910 cells were transfected with S100A11 or control shRNA using Lipofectamine^®^ LTX with Plus^™^ reagent (Invitrogen Life Technologies, Carlsbad, CA, USA). The stable cell clones were isolated using Geneticin^®^ Selective Antibiotic (G418 Sulfate; Gibco Life Technologies, Beijing, China).

### Western blot analysis

Total protein was extracted using radioimmunoprecipitation assay lysis buffer and the protein concentration was determined using a bicinchoninic acid assay (Sigma-Aldrich). Subsequently, 50 mg total protein was resolved using SDS-PAGE and the separated protein was transferred onto a polyvinylidene fluoride membrane (EMD Millipore, Billerica, MA, USA). The membrane was blocked in 5% non-fat milk for 1 h and then incubated with primary antibodies overnight at 4°C. After washing three times with phosphate-buffered saline with Tween-20, the membrane was incubated with peroxidase-conjugated AffiniPure goat anti-mouse (ZB-2305) and goat anti-rabbit (ZB-2301) immunoglobulin G secondary antibodies (1:3,000; Zhongshan Jinqiao Biotech, Co., Ltd., Beijing, China) for 1 h at room temperature. Bands were detected with an enhanced chemiluminescence detection kit (Applygen Technologies, Inc., Beijing, China) and the densitometry of each band was analyzed with ImageJ software.

### Cell growth assay

Cells were seeded into a 24-well plate at 1×10^4^ cells/well and incubated in RPMI 1640 medium overnight. Cells in the plate were trypsinized (Sigma-Aldrich and counted using a hemocytometer (Sigma-Aldrich) every day for seven days. The experiments were repeated three times.

### MTT assay

Cell growth rate was determined by an MTT assay. Briefly, 2×10^3^ cells/well were seeded into a 96-well plate. Subsequently, 20 μl MTT solution (5 mg/ml; Sigma-Aldrich) was added to each well and the cells were further incubated at 37°C in 5% CO_2_ for 4 h. Next, the RPMI 1640 medium was removed and dimethyl sulfoxide was added to the wells to dissolve the colored formazan crystals produced by MTT. Finally, the optical density (OD) value was measured at 490 nm using a Bio-Rad 2550 EIA Reader (Bio-Rad Laboratories, Inc., Hercules, CA, USA).

### Soft agar assay

A soft agar assay was used to examine the anchorage-independent growth of ovarian cancer cells. Cells at a density of 0.5×10^3^ cells/ml were suspended in RPMI 1640 medium supplemented with 10% FBS and 0.3% agarose. The cell suspension was added to a base layer formed by culture medium containing 0.6% agarose. After 14 days, the colony number of each well was counted under an inverted microscope (Olympus X71; Olympus Corporation, Tokyo, Japan) at ×100 magnification.

### Invasion assay

A 24-well Transwell^®^ plate was obtained from Corning, Inc. (Costar^®^; Corning, NY, USA), and the cell inserts were coated with Matrigel™ (BD Biosciences, Franklin Lakes, NJ, USA). Cells (1.0×10^5^) in RPMI 1640 medium were plated in the upper chambers, while the lower chambers were filled with RPMI 1640 medium containing 20% FBS as a chemoattractant. The cells were allowed to invade for 24 h, at 37°C and 5% CO_2_. Cells in the upper chambers were removed with a cotton swab and the filters were stained with eosin. Next, five random fields were observed and the cell numbers were counted under an inverted microscope (Olympus X71) at ×100 magnification.

### Migration assay

A migration assay was performed with a 24-well Transwell^®^ plate. Cells were suspended in RPMI 1640 medium and adjusted to a concentration of 5.0×10^5^ cells/ml. A 100-μl cell suspension was plated in the upper chambers, and RPMI 1640 medium containing 20% FBS was filled in the lower chambers as a chemoattractant. After 24 h, the non-migrated cells were removed with a cotton swab and the filters were stained with eosin. Finally, the number of migrated cells was counted under an inverted microscope (Olympus X71) at ×100 magnification.

### Statistical analysis

Experiments were performed a minimum of three times and the results are presented as the mean ± standard deviation. The Student’s t-test was used for statistical analysis, where P<0.05 was considered to indicate a statistically significant difference.

## Results

### Expression levels of S100A11 are elevated in ovarian cancer cells

**Figure 1:**
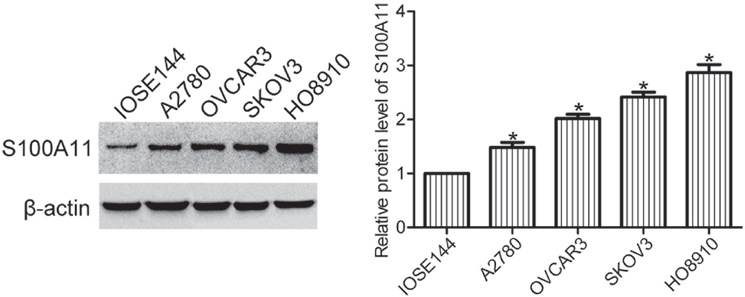
Protein expression levels of S100A11 in the different ovarian cell lines (ovarian surface epithelial cell line, IOSE144; ovarian cancer cell lines, A2780, OVCAR3, SKOV-3 and HO8910) were determined by western blot analysis. ^*^P<0.05, vs. IOSE144 cells.

Expression levels of S100A11 in human ovarian surface epithelial cells (IOSE144) and ovarian cancer cells (A2780, OVCAR3, SKOV3 and HO8910) were determined using western blot analysis. The expression levels of S100A11 were markedly increased in the ovarian cancer cell lines when compared with the ovarian surface epithelial cell line (Fig. 1), indicating that S100A11 may play a role in the progression of ovarian cancer.

### Expression of S100A11 in HO8910 cells is silenced by shRNA

**Figure 2:**
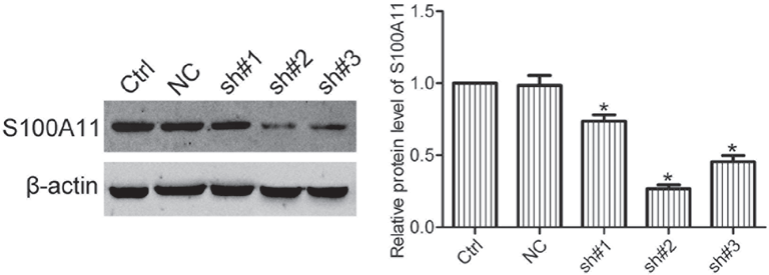
Stable knockdown of S100A11 by short hairpin (sh)RNA in the HO8910 cells. Following transfection with S100A11 shRNA, stable cell clones were isolated by G418 selection. Western blot analysis was performed to examine the expression of S100A11 in Ctrl, NC and S100A11 shRNA cells (sh#1, sh#2 and sh#3). ^*^P<0.05, vs. Ctrl and NC cells. Ctrl, mock-transfected control; NC, negative control.

In order to silence the expression of S100A11 in ovarian cancer cells, HO8910 cells were transfected with S100A11 shRNA (sh), and three stable cell clones (sh#1, sh#2 and sh#3) were isolated by G418 selection. Western blot analysis showed that sh#2 cells significantly repressed the expression of S100A11 in HO8910 cells (Fig. 2). Therefore, sh#2 cells were used in the following experiments.

### Knockdown of S100A11 inhibits the growth of ovarian cancer cells

**Figure 3:**
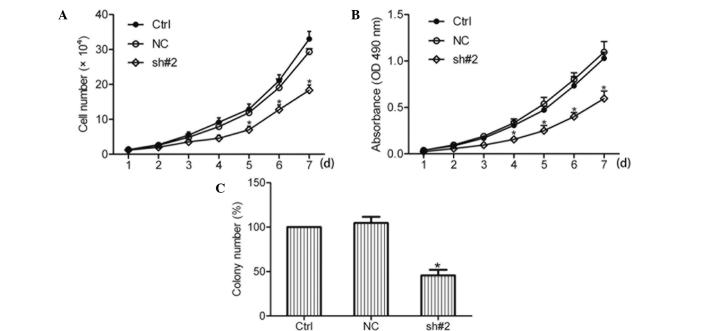
Effect of S100A11 knockdown on ovarian cancer cell growth. (A) Cell growth and (B) MTT assays were performed to compare the growth of Ctrl, NC and sh#2 cells. ^*^P<0.05,. (C) Soft agar assay was performed to compare the anchorage-independent growth of Ctrl, NC and sh#2 cells. ^*^P<0.05, vs. Ctrl and NC cells. Ctrl, mock-transfected control; NC, negative control; OD, optical density; sh, short hairpin.

Effects of S100A11 knockdown on the growth of ovarian cancer cells were examined using a cell growth assay, which revealed that knockdown of S100A11 inhibited the growth of ovarian cancer cells (Fig. 3A). In order to confirm this result, an MTT assay was performed and the results were found to be consistent with the cell growth assay (Fig. 3B). Furthermore, the effects of S100A11 knockdown on the anchorage-independent growth of ovarian cancer cells were examined using a soft agar assay. The results revealed that knockdown of S100A11 decreased the number of HO8910 cell colonies (Fig. 3C). Collectively, these results indicated that S100A11 was involved in the regulation of ovarian cancer cell growth.

### Knockdown of S100A11 suppresses cell invasion and migration

**Figure 4:**
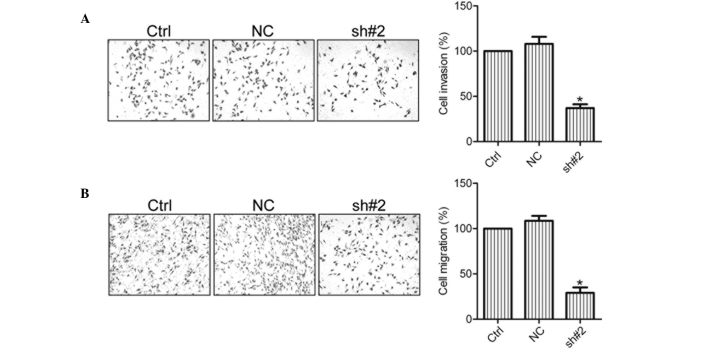
Effect of S100A11 knockdown on ovarian cancer cell (A) invasion and (B) migration in Ctrl, NC and sh#2 cells. Invaded/migrated cells were stained with eosin, and five random fields were observed under a microscope at ×100 magnification. ^*^P<0.05, vs. Ctrl and NC cells. Ctrl, mock-transfected control; NC, negative control; sh, short hairpin.

In order to determine whether S100A11 was associated with the invasion and migration of ovarian cancer cells, invasion and migration assays were conducted with mock-transfected control (Ctrl), negative control (NC) and S100A11 shRNA cells (sh#2). The results demonstrated that the invasion and migration activity of HO8910 cells was greatly suppressed in the sh#2 cells when compared with the Ctrl and NC cells (Fig. 4), indicating that S100A11 was able to promote the invasion and migration of ovarian cancer cells.

### Knockdown of S100A11 decreases the expression levels of Snail, but increases those of E-cadherin

**Figure 5:**
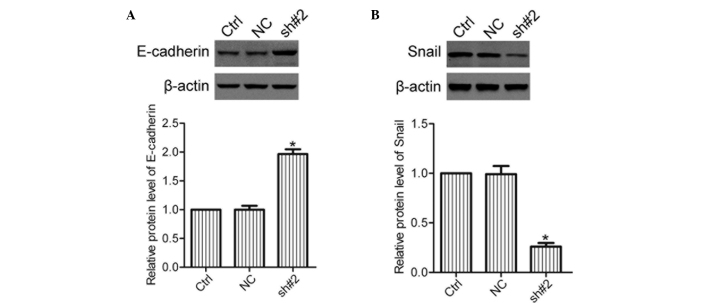
Effect of S100A11 knockdown on the expression levels of (A) E-cadherin and (B) Snail in Ctrl, NC and sh#2 cells, as examined by western blot analysis. ^*^P<0.05, vs. Ctrl and NC cells. Ctrl, mock-transfected control; NC, negative control; sh, short hairpin.

Snail and E-cadherin are important epithelial-mesenchymal transition (EMT) markers that are essential for the invasion and metastasis of cancer (11). The expression levels of E-cadherin and Snail in the Ctrl, NC and sh#2 cells were analyzed using western blot analysis. The results revealed that knockdown of S100A11 caused an increase in E-cadherin and a decrease in Snail protein expression levels (Fig. 5), suggesting that S100A11 affected the expression of E-cadherin and Snail in ovarian cancer cells.

## Discussion

S100A11 is a member of the S100 protein family, and is widely expressed in human tissues. Overexpression of S100A11 has been identified in a variety of human cancer types and elevated S100A11 expression is closely associated with tumor progression (12). Increased levels of S100A11 have been reported in ovarian cancer tissues, as compared with normal ovarian epithelial tissues (13). In the present study, the expression levels of S100A11 were found to be significantly increased in the ovarian cancer cell lines. Thus, S100A11 may play an important role in the progression of ovarian cancer.

S100A11 has a number of contrasting roles in the regulation of tumor growth. A previous study found that S100A11 promoted the proliferation of lung cancer cells (14). In addition, silencing S100A11 expression has been shown to reduce the anchorage-independent growth of papillary thyroid carcinoma cells (5). However, S100A11 is also reported to be a tumor suppressor in hepatocellular carcinoma cells and epidermal keratinocytes (15,16). The role of S100A11 in ovarian cancer has not been fully characterized. The results of the present study demonstrated that knockdown of S100A11 inhibited the proliferation and anchorage-independent growth of HO8910 cells, suggesting that S100A11 contributes to the growth of ovarian cancer cells.

A previous study reported that overexpression of S100A11 is associated with the metastasis of cancer (17). In addition, experimental studies have shown that S100A11 is a migration-related protein in laryngeal squamous cell carcinoma (18), and is involved in the invasion of hepatocellular carcinoma cells (19). A recent study showed that S100A11 is required for the survival of invasive cancer cells (20). In the present study, knockdown of S100A11 was found to suppress the invasion and migration of HO8910 cells, indicating that S100A11 may be involved in the regulation of ovarian cancer cell invasion and migration.

EMT plays a key role in the invasion and metastasis of ovarian cancer (21), and E-cadherin and Snail are crucial EMT cancer markers (22). E-cadherin is a transmembrane protein that regulates cell-cell adhesion, while Snail is a transcriptional repressor of E-cadherin gene expression (23). S100A4 has been reported to enhance pancreatic cancer cell invasion via the regulation of E-cadherin expression (24), and overexpression of S100A6 has been reported to result in the downregulation of E-cadherin (25). However, whether S100A11 affects the expression levels of E-cadherin and Snail has not yet been investigated. The present study demonstrated that knockdown of S100A11 increased the expression of E-cadherin and decreased the expression of Snail in ovarian cancer cells. In addition, knockdown of S100A11 inhibited the invasion and migration of ovarian cancer cells, indicating that S100A11 may promote ovarian cancer cell invasion and migration via the regulation of E-cadherin and Snail expression.

In conclusion, S100A11 is overexpressed in ovarian cancer cells and the knockdown of S100A11 suppresses the growth, invasion and migration of ovarian cancer cells. Thus, inhibition of S100A11 may provide a promising approach to the treatment of ovarian cancer.
